# A Rare Case of Multiple Recurrent Parasitic Leiomyomas Managed Surgically Without Additional Chemical Estrogen Suppression

**DOI:** 10.1155/crom/5552760

**Published:** 2026-10-21

**Authors:** Claire Baptiste, Elise N. Everett, Hibba tul Rehman, Alexandra Fenton, Bronwyn Bryant, Jessica Cintolo-Gonzalez

**Affiliations:** ^1^ The Robert Larner College of Medicine at the University of Vermont Burlington Vermont USA; ^2^ Department of Obstetrics Gynecology and Reproductive Sciences The University of Vermont Medical Center Burlington Vermont USA; ^3^ Department of Medicine Division of Hematology/Oncology Department of Medicine University of Maryland School of Medicine Baltimore Maryland USA umd.edu; ^4^ Department of Pathology and Laboratory Medicine The Robert Larner College of Medicine at the University of Vermont Burlington Vermont USA lhsc.on.ca; ^5^ Department of Surgery Division of Surgical Oncology Rutgers Cancer Institute New Brunswick New Jersey USA

**Keywords:** abdominal mass, estrogen suppression, morcellation, oophorectomy, parasitic leiomyoma, recurrent fibroid

## Abstract

Parasitic leiomyomas (PMs) are rare extrauterine benign smooth muscle tumors that can arise spontaneously or develop iatrogenically following prior uterine surgery, particularly with morcellation of uterine tissue. We present a case of a 49‐year‐old woman who developed multiple recurrent PMs involving the abdominal cavity, appendix, and abdominal wall over several years following laparoscopic myomectomy with morcellation and subsequent hysterectomy. At her most recent intra‐abdominal recurrence, she underwent a bilateral oophorectomy at the time of tumor resection. This case highlights the potential for delayed recurrence of parasitic myomas even years after hysterectomy and supports the importance of clinical vigilance and imaging in symptomatic patients with prior morcellation. It also supports consideration of and further study of the role of estrogen suppression in preventing recurrence.

## 1. Introduction

Parasitic leiomyomas (PMs) are rare extrauterine benign smooth muscle tumors that can arise spontaneously outside the uterus, typically within the peritoneum or omentum or develop iatrogenically following prior uterine surgery, with or without morcellation [1–8]. They can also form spontaneously when a pedunculated subserosal fibroid loses its uterine blood supply and establishes a new blood supply from adjacent structures, allowing it to grow independently. The clinical presentation can vary, with symptoms ranging from asymptomatic masses to abdominal pain, bowel obstruction, or urinary symptoms, depending on the location and size of the tumor. Because of their rarity, optimal preventative strategies and postoperative surveillance are not standardized. Hormonal suppression therapy and surgical adjuncts such as oophorectomy or omentectomy have been variably reported, but their roles remain uncertain. This case contributes to ongoing efforts to clarify which patients may benefit from additional preventive measures following resection.

## 2. Case Presentation

In May 2019, a 42‐year‐old premenopausal female with a body mass index (BMI) of 37 presented for evaluation at her gynecology office with pelvic pain and abnormal uterine bleeding despite having an IUD. Her surgical history was notable for a prior myomectomy with power morcellation of fibroids in 2012 at age 36 and a diagnostic laparoscopy for recurrent fibroids in 2018 at age 41. She was taken back to the operating room for hysterectomy for abnormal uterine bleeding and surgical findings revealed multiple parasitic leiomyomatous implants throughout the abdominal cavity, including the appendix. She underwent exploratory laparotomy, total abdominal hysterectomy with ovarian preservation, appendectomy, resection of peritoneal implants, and debulking of the extrauterine leiomyomas. Pathology confirmed benign leiomyomas; a low‐grade appendiceal mucinous neoplasm was also found incidentally, confined to the appendiceal wall and removed with negative margins. No further treatment was required.

In November 2020, at age 44, she developed a laparoscopic port‐site abdominal wall mass, which was excised and diagnosed as smooth muscle neoplasm, consistent with leiomyoma, without atypical features in sections examined (Figure 1). She remained well until May 2022, when she presented with right lower quadrant pain and changes in bowel habits. CT imaging demonstrated an isolated 4.2 cm mass along the proximal ascending colon (Figure 2). The patient desired oophorectomy at the time of surgical resection. A multidisciplinary team including surgical oncology, medical oncology, and gynecologic oncology reviewed the role of estrogen in the recurrence of parasitic leiomyomas and discussed the risks and benefits of surgical menopause, chemically induced menopause, and further hormone suppression with an aromatase inhibitor to lower endogenous estrogen levels and potentially reduce the risk of recurrence. After a shared‐decision making process, the patient and surgical team agreed upon surgical oophorectomy concurrent with surgical excision of the mass.

**Figure 1:**
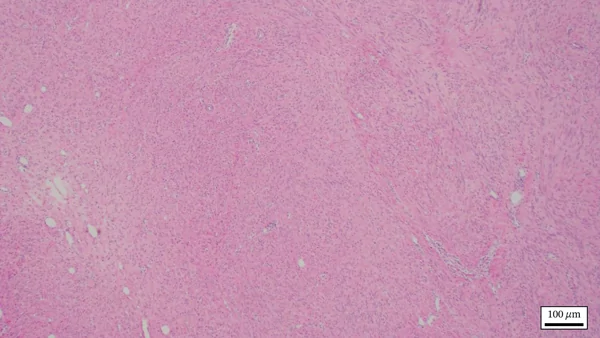
Histologic section of port‐site abdominal wall mass excised in 2020 showing a smooth muscle neoplasm consistent with conventional leiomyoma; no atypical features were identified in sections examined (hematoxylin and eosin, 400x).

**Figure 2:**
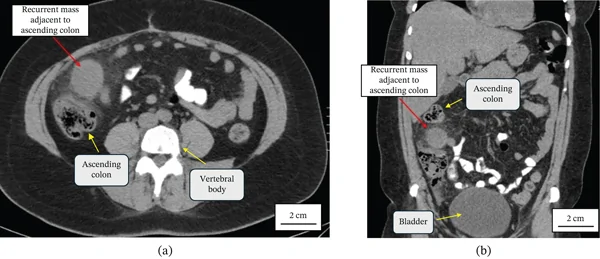
CT of the abdomen and pelvis without contrast (2022) demonstrating a solid mass, consistent with recurrent parasitic leiomyoma in the (a) axial and (b) coronal planes, adjacent to the ascending colon in the right lower quadrant.

The patient underwent exploratory laparotomy, bilateral oophorectomy, partial omentectomy, and excision of the mass. Findings at the time of surgery demonstrated that her mass was adjacent to but not invading the colon, and embedded in the greater omentum. Pathology confirmed an infarcted smooth muscle neoplasm, consistent with recurrent leiomyoma. No significant cytologic atypia, mitotic activity, or coagulative necrosis was identified. Ovarian pathology revealed normal physiologic structures and surface adhesions. No adjuvant hormonal therapy was administered.

Postoperatively, she experienced vasomotor menopausal symptoms, including hot flashes and night sweats, which were partially managed with venlafaxine 150 mg daily. She initially reported stress urinary incontinence with coughing and sneezing but denied pelvic pain or dysuria. Surveillance CT at 6, 12, and 24 months showed no evidence of disease, and she remains disease‐free 4.5 years postoperatively. Despite the favorable disease‐free interval and avoidance of repeat surgery, she experienced persistent, at times severe, vasomotor symptoms following oophorectomy. Because of the diagnosis of PM, which was the rationale behind her oophorectomies, she has avoided hormone replacement therapy for her vasomotor symptoms. However, she recently initiated therapy with fezolinetant, a neurokinin 3 receptor (NK3R) antagonist and nonhormonal option for managing vasomotor symptoms, with subsequent improvement in both symptoms and quality of life. A visual summary of this clinical timeline is provided in Figure 3.

**Figure 3:**
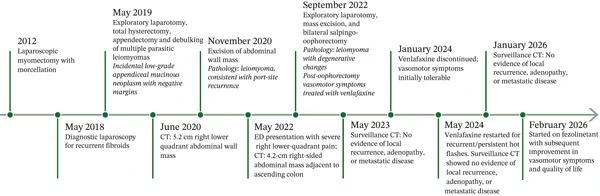
Timeline of recurrent leiomyoma and surgical management from 2012 to 2026.

## 3. Discussion

PMs, although benign, represent a rare but clinically significant complication following uterine surgery, particularly in the context of laparoscopic morcellation [1, 2]. Recurrence can occur even after complete surgical excision, highlighting the importance of vigilant long‐term surveillance ^3, 4.^

Hormonal influences are central to leiomyoma growth, with estrogen and progesterone receptors commonly expressed in these tumors. Although this is true for conventional leiomyomas, which are more common in premenopausal patients, there is evidence that hormones can drive PMs. Most of the literature for PMs consists of case reports or case series. Although there are larger studies that report on recurrence risk of conventional leiomyoma, particularly after myomectomy, it is unclear the extent to which this data can be extrapolated for management of PMs. Obesity may further influence this hormonal milieu, as increased adipose tissue contributes to peripheral aromatization of androgens into estrogen, potentially leading to higher circulating estrogen levels and promoting hormone‐responsive tumor growth [5]. Report of the impact of BMI as a modifiable risk factor for uterine leiomyoma is variable [9].

Huang and colleagues developed mouse models that demonstrated upregulation of estrogen receptor *α*, progesterone receptor, and markers of angiogenesis in PMs compared with uterine myomas. In a mouse model, estrogen depletion decreased implantation of PMs suggesting hormone manipulation as a therapeutic strategy [6]. Surgical strategies such as oophorectomy can reduce circulating hormone levels and may contribute to decreased recurrence risk [6, 7]. Reports also suggest that aromatase inhibitors may be considered for patients with contraindications to oophorectomy or those with residual disease, though evidence remains limited [8]. Most patients are reported to be treated with resection of the recurrences alone, though there is a case series of two patients treated with 3 months of adjuvant GnRH‐*α* [10]. This preclinical and clinical data are limited but suggests that further study about the role of hormonal suppression in these patients is warranted.

Genetic predisposition may also play a role, with some patients more susceptible to disseminated leiomyomatosis, as suggested by observational studies at tertiary centers [11, 12]. In addition, increasing attention has been given to fumarate hydratase (FH)–deficient leiomyomas, which occur sporadically and in association with hereditary leiomyomatosis and renal cell carcinoma (HLRCC), a syndrome that confers increased risk for aggressive RCC [13]. Defining the histopathologic features of FH‐deficient leiomyomas has led to the development of screening algorithms that start with recognizing key histopathologic features followed by immunohistochemical evaluation for FH‐deficiency. This approach, particularly in younger patients, can help identify patients who may benefit from genetic evaluation and renal cancer surveillance [14]. Although this entity was not widely recognized at the time of this patient′s initial surgery in 2012, contemporary evaluation of unusual, recurrent, or disseminated leiomyomas increasingly considers FH deficiency. In this case, available pathology has not demonstrated histologic features suggestive of FH‐deficient leiomyoma such as more epithelioid nuclei, prominent eosinophilic nucleoli, perinucleolar halos, and staghorn vasculature. Referral for genetic counseling and testing may also be considered based on personal and family history including cutaneous leiomyomata, aggressive uterine leiomyomata, and renal tumors [15]. This option was discussed with the patient given her young age at presentation and multiple recurrences, but due to lack of other personal or familial risk factors and reassuring pathology evaluation, the patient declined a referral for genetic testing.

The decline in the use of uncontained power morcellation—in part due to cases of PM, leiomyomatosis, and even leiomyosarcoma [12, 16]—has also likely contributed to a decrease in iatrogenic PMs. However, spontaneous and recurrent cases continue to occur, emphasizing the need for clinicians to maintain a high index of suspicion in patients with relevant surgical history presenting with abdominal pain, mass effect, or bowel or urinary symptoms. Patient‐centered management, including individualized discussion of treatment for menopausal symptoms, is essential to balance quality of life with recurrence risk [17]. Although hormone replacement may improve quality of life, the benefits may be outweighed by concern for recurrent disease. There are multiple alternatives to hormone therapy for managing menopause particularly as hormone replacement is contraindicated for many patients including those with history of hormone receptor positive breast cancer [18]. Patients with genitourinary symptoms following surgical menopause and have a contraindication to systemic estrogen therapy may benefit from vaginal estrogen therapy, which should be considered through shared decision‐making in coordination with the surgical and gynecologic care team. In this case, the patient had elected for oophorectomy because of multiple recurrences and short disease‐free intervals between her more recent recurrences. An alternative could have been observation of this isolated recurrence or surgical resection alone. Another, more aggressive option would be surgical resection followed by adjuvant endogenous estrogen suppression with an aromatase inhibitor, with potential risks of significant vasomotor symptoms and reduced quality of life. Currently her disease‐free interval is 4.5 years. This case highlights individualized clinical decision‐making accounting for each patient′s risk factors for recurrence, clinical course, and quality of life, as well as the importance of shared decision making between the multi‐disciplinary team and the patient.

## 4. Conclusion

This case illustrates that recurrent PMs can develop years after initial surgery and may require multiple surgical interventions for definitive management. Complete surgical excision combined with postoperative surveillance can result in durable disease control, even in the absence of hormone manipulation. However, surgical or chemical hormone manipulation to reduce endogenous estrogen levels may have a role in decreasing recurrence. Clinicians should be aware of the potential for delayed recurrence and engage in individualized counseling regarding hormonal management, risk factors for a genetic predisposition, and preventive strategies, as well as the risks and side effects associated with these strategies.

## Funding

No funding was received for this manuscript.

## Disclosure

The lead and corresponding author, Dr. Jessica Cintolo‐Gonzalez, affirms that this manuscript is an honest, accurate, and transparent account of the study being reported; that no important aspects of the study have been omitted; and that any discrepancies from the study as planned (and, if relevant, registered) have been explained. All authors have read and approved the final version of the manuscript. Dr. Jessica Cintolo‐Gonzalez had full access to all of the data in this study and takes complete responsibility for the integrity of the data and the accuracy of the data analysis.

## Ethics Statement

The authors have nothing to report.

## Consent

Written consent was obtained for the publication of deidentified information and case details.

## Conflicts of Interest

The authors declare no conflicts of interest.

## Data Availability

The data that supports the findings of this study are available from the corresponding author upon reasonable request. The data are not publicly available due to privacy or ethical restrictions.
